# ATPase copper transporting beta attenuates malignant features with high expression as an indicator of favorable prognosis in breast cancer

**DOI:** 10.1007/s12282-025-01705-7

**Published:** 2025-05-02

**Authors:** Ikumi Soeda, Masahiro Shibata, Takahiro Inaishi, Takahiro Ichikawa, Kayoko Sugino, Emi Kanaya, Mitsuro Kanda, Masamichi Hayashi, Norikazu Masuda

**Affiliations:** 1 Department of Breast and Endocrine Surgery, Nagoya University Graduate School of Medicine, 65 Tsurumai-cho, Showa-ku, Nagoya, 466-8550 Japan; 2 Department of Surgery, Nagoya Ekisaikai Hospital, 4-66, Shonen-cho, Nakagawa-ku, Nagoya, 454-8502 Japan; 3 Department of Surgery, Komaki City Hospital, 1-20, Joubushi, Komaki, Aichi 485-8520 Japan; 4 Department of Surgery and Science, Faculty of Medicine, Academic Assembly, University of Toyama, 2630 Sugitani, Toyama-shi, Toyama 930-0194 Japan; 5 Department of Gastroenterological Surgery, Nagoya University Graduate School of Medicine, 65 Tsurumai-cho, Showa-ku, Nagoya, 466-8550 Japan

**Keywords:** ATPase copper transporting beta, Breast cancer, Estrogen receptor, Prognostic marker

## Abstract

**Background:**

ATPase copper transporting beta (ATP7B) functions as a copper-transporting ATPase that ejects copper from cells. Although high expression of ATP7B has been reported to increase cisplatin resistance, its role in breast cancer (BC) remains unclear. This study aimed to elucidate the function of ATP7B in BC cells and its significance in patients with BC.

**Methods:**

The mRNA and protein expression levels of ATP7B were evaluated in BC and non-cancerous mammary cell lines. Polymerase chain reaction (PCR) array analysis was conducted to determine the correlation between *ATP7B* and 84 cancer-related genes. *ATP7B* knockdown was performed using small interfering RNA, and cell proliferation, invasiveness, and migration were analyzed. The associations between the mRNA and protein expression of ATP7B and clinicopathological factors were also investigated in 156 patients with BC.

**Results:**

*ATP7B* was found to be highly expressed in estrogen receptor-positive and human epidermal growth factor receptor 2-positive BC cell lines. PCR array analysis revealed a significant correlation between the expression level of *ATP7B* and those of *cadherin 1, estrogen receptor 1,* and *MET proto-oncogene*. *ATP7B* knockdown significantly increased the proliferation, invasiveness, and migration of MDA-MB-361 and MDA-MB-415 cells. Patients with high ATP7B expression at the mRNA and protein levels experienced favorable prognoses. In addition, ATP7B expression level was identified as an independent prognostic factor in multivariate analysis.

**Conclusions:**

ATP7B is involved in promoting anti-cancer activities of tumor suppressors in BC cells across different subtypes and is considered a prognostic marker for BC.

**Supplementary Information:**

The online version contains supplementary material available at 10.1007/s12282-025-01705-7.

## Introduction

Breast cancer (BC) is the most common cancer in women worldwide [1]. BC is classified via immunohistochemical analysis using conventional targets, including the estrogen receptor (ER), progesterone receptor (PgR), and human epidermal growth factor receptor 2 (HER2), and is treated according to the classification. Although several therapeutic agents are available and are still being developed, the 5-year survival rate of patients with distant metastases is as low as 27%, indicating that curing BC remains difficult [1]. Therefore, new biomarkers and therapeutic target molecules are needed to improve the prognosis of patients with BC.

ATPases are a general term for enzymes that hydrolyze the phosphate bonds of adenosine triphosphate and convert the energy obtained by hydrolysis to other tasks. The function of P-type ATPases is the transport of various ions and lipids [2]. One of the P-type ATPases, copper-transporting ATPase α/β (ATP7A/ATP7B), is involved in the intracellular transport and homeostasis of copper. Mutations in ATP7B are known to cause Wilson’s disease as biliary excretion of copper is inhibited due to these mutations [3]. In malignant tumors, high expression of *ATP7A* or *ATP7B* increases cisplatin resistance [4, 5]. Recently, *ATP7A* and *ATP7B* have also attracted attention owing to their potential involvement in cuproptosis [6]. ATP7A contributes to cisplatin resistance by regulating miRNAs in BC cell lines [7]. High expression of *ATP7B* was found to be associated with decreased survival in patients with colorectal and lung squamous cell carcinomas and increased survival in those with renal clear cell carcinoma, low-grade glioma, and thyroid cancer [8]. Although BC cell lines expressing ATP7B have been reported to be more resistant to cisplatin than those without its expression [9], the functional role of ATP7B in BC and its impact on patients have not been reported to date. This study aimed to elucidate the role of ATP7B in BC cells and determine the significance of its expression in patients with BC.

## Materials and methods

### Ethics

This study was conducted in accordance with the principles of the Declaration of Helsinki and was approved by the Institutional Review Board and Ethics Committee of Nagoya University Hospital (approval no.: 2019-0028). All patients provided written informed consent for the use of clinical specimens and data.

### Sample collection

A total of 13 BC cell lines (BT-20, BT-474, BT-549, HCC1419, HCC1954, Hs578T, MCF7, MDA-MB-231, MDA-MB-361, MDA-MB-415, MDA-MB-468, SK-BR-3, and ZR-75-1) and two non-cancerous breast epithelial cell lines (MCF-10A and MCF-12A) were obtained. BT-549, HCC1419, HCC1954, and Hs578T cell lines were purchased from the Japanese Collection of Research Bioresources Cell Bank (Osaka, Japan). BT-474, MCF7, and MCF-12A cells were kindly provided by Prof. David Sidransky of Johns Hopkins University (Baltimore, MD, USA). Other cell lines were purchased from American Type Culture Collection (Manassas, VA, USA). All cells were stored using a cell preservation solution (Cell Banker; Mitsubishi Chemical Medicine Corporation, Tokyo, Japan) at − 80 °C, cultured in RPMI-1640 (Sigma-Aldrich, St. Louis, MO, USA) supplemented with 10% fetal bovine serum (FBS), and incubated in an atmosphere of 5% carbon dioxide at 37 °C [10, 11].

BC and non-BC tissues were collected from 156 patients who were pathologically diagnosed with BC and underwent breast surgery at Nagoya University Hospital between March 2002 and May 2007. Noncancerous tissue was collected at least 3 cm from the edge of the tumor. All harvested tissues were immediately cut into approximately 1.5 mm sections and stored at − 80 °C [12]. The BC stages were classified using the Union for International Cancer Control (UICC) staging system (8th edition). Perioperative adjuvant therapy was determined based on the patient’s general condition, pathology, subtype classification, and shared decision-making between the attending physician and patient [12].

### Quantitative real-time reverse transcription polymerase chain reaction (RT-qPCR)

*ATP7B* mRNA expression levels were determined using RT-qPCR. RNA was extracted from the BC and non-cancerous specimens collected from 156 patients and each cell line (8.0 × 10^6^ cells per cell line). cDNA was synthesized as previously described [10, 11]. *Glyceraldehyde-3-phosphate dehydrogenase* (*GAPDH*) mRNA levels were quantified to normalize the expression levels. The specific primers for each gene were as follows: *ATP7B*, forward 5′-AGATCACAGCCAGAGAAGGG-3′ and reverse 5′-GCCAACATTGTCAAAAGCAA-3′, which generated a 110-bp product; and *GAPDH*, forward 5′-GAAGGTGAAGGTCGGAGTC-3′ and reverse 5′-GAAGATGGTGATGGGATTTC-3′, which generated a 226-bp product [12]. qRT-PCR was performed using an ABI StepOnePlus real-time PCR System (Applied Biosystems, Foster City, CA, USA), as previously described [10, 11]. The mRNA expression level of* ATP7B* was determined by dividing the value of each sample by the corresponding* GAPDH* value [10, 11].

### PCR array analysis

To determine the correlation between the expression levels of *ATP7B* and 84 cancer-related genes in BC cell lines, PCR array analysis was performed using the RT^2^ Profiler PCR Array Human Oncogenes & Tumor Suppressor Genes (Qiagen, Hilden, Germany), according to the manufacturer’s protocol. The relative expression levels of these genes in each sample were determined by dividing the relevant values by their corresponding *GAPDH* values.

### ATP7B knockdown using *ATP7B*-specific small interfering RNAs (siRNAs)

MDA-MB-361 and MDA-MB-415 cell lines were transfected with siRNA specific for *ATP7B* (designated “si*ATP7B*”: 5′-CCAAUUGAUAUUGAGCGGUUATT-3′; Hokkaido System Science, Sapporo, Japan) to knockdown *ATP7B*. Fluorescein-labeled AccuTarget negative control siRNA (siControl, Cosmo Bio Co. Ltd., Tokyo, Japan) served as the nontargeting siRNA, designated “siControl.” BC cells were transfected with siRNAs via electroporation using the Neon System (Thermo Fisher Scientific, Waltham, MA, USA). The untransfected cells were electropulsed without siRNA. After the electric pulse, cells were cultured in antibiotic-free RPMI-1640 with 10% FBS for 72 h. The knockdown efficiency was determined using qRT-PCR and western blotting.

### Western blotting

Western blotting was performed using a Wes Simple Western System (ProteinSimple, San Jose, CA, USA), according to the manufacturer’s instructions. Cultured cells were lysed in RIPA lysis buffer and the lysate was stored at − 30 °C. Protein concentrations were measured using the BCA protein assay kit (Thermo Fisher Scientific). Protein samples were aliquoted into assay plates and automatically detected in individual capillaries. Anti-ATP7B antibody (1:250 dilution; cat. no. ab124973; Abcam, Cambridge, UK) and anti-beta-actin antibody (1:250 dilution; cat. no. ab6276; Abcam, Cambridge, UK) were used as the primary antibodies. Streptavidin Western horseradish peroxidase and anti-mouse or anti-rabbit secondary antibodies (ProteinSimple, San Jose, CA, USA) were selected based on the corresponding primary antibody [13, 14].

### Proliferation assay

Cell proliferation was evaluated using the Cell Counting Kit-8 (CCK-8) (Dojindo Molecular Technologies, Inc., Kumamoto, Japan). MDA-MB-361 (1.0 × 10^4^ cells per well) and MDA-MB-415 (1.0 × 10^4^ cells per well) cells transfected with si*ATP7B* or siControl, or untransfected cells were seeded into 96-well plates with RPMI-1640 containing 2% FBS. Each sample was added to six wells and cultured for the indicated time periods. The optical density (450 nm) of each well was measured 2 h after the addition of 10 µL of CCK-8 solution from the start of seeding to day 5 post-seeding [12].

### Invasiveness assay

Cellular invasiveness was determined using BioCoat Matrigel Invasion Chambers (pore size 8‑μm; Corning Inc., Corning, NY, USA), according to the manufacturer’s protocol. After transfection, MDA-MB-361 (3 × 10^5^ cells per well) and MDA-MB-415 (3 × 10^5^ cells per well) cells were suspended in serum-free RPMI-1640 and seeded into the upper chambers. RPMI-1640 medium supplemented with 20% FBS was added to the bottom row of the wells. After 72 h of incubation, cells on the membrane surfaces were fixed and stained with Diff Quik (cat. no. 16920; Sysmex, Kobe, Japan) solutions I and II for 5 s at room temperature. Cells on the membrane were counted in 10 randomly selected fields of view using an upright microscope (Olympus Corporation) at ×100 magnification [12].

### Migration assay

The migration of MDA-MB-361 and MDA-MB-415 cells was determined using a wound-healing assay. After transfection, MDA-MB-361 (5.6 × 10^4^ cells per well) and MDA-MB-415 (5.6 × 10^4^ cells per well) cells were seeded in each well of Culture-Insert 2 Well (Ibidi, Martinsried, Germany), which were attached to 24-well plate using RPMI-1640 containing 10% FBS. After 24 h, the insert was removed and replaced with FBS-free RPMI-1640 medium and the 24-well plate was placed in an IncuCyte SX5 analysis system (Sartorius, Gottingen, Germany). The same sites were automatically photographed at 0, 12, 24, 48, and 72 h. Wound widths were measured 20 times per well at 100-μm intervals [12].

### Immunohistochemistry

Of the 156 patients mentioned above, specimens from 152 were available for immunohistochemical analysis. Formalin‑fixed, paraffin‑embedded sections (4‑μm thick) were constructed from blocks of resected specimens. The ATP7B rabbit polyclonal antibody (1:500 dilution) (cat. no. NB100-360; Novus biologicals, LLC., Centennial, CO, USA) was used for immunohistochemistry, and sections were incubated overnight at 4 °C. The EnVision + System- HRP Labelled Polymer Anti-Rabbit (cat. no. K4003; Dako North America Inc. Carpinteria, CA, USA) was used as the secondary antibody and the sections were incubated for 30 min at room temperature. The cancerous area of each section was observed under an upright light microscope (Olympus Corporation; ×40, ×100, and ×400 magnification). The intensity of staining (IS) in the cytoplasm of cancer cells was evaluated and divided into four levels, ranging from 0 (negative) to 3 (strong). The percentage of staining (PS) was evaluated for whole cancers and divided into 11 levels, ranging from 0 to 100% in 10% increments. The IP score was assigned by multiplying the IS by the PS.

### Public datasets of BC cell lines and patients

The mRNA expression levels of *ATP7B* in 59 BC cell lines were obtained from the Cancer Cell Line Encyclopedia (CCLE) database (https://sites.broadinstitute.org/ccle/). The data were accessed on August 28, 2022. The Kaplan–Meier plotter website (http://kmplot.com/analysis/index.php?p=background) was used to analyze relapse-free survival (RFS) and overall survival (OS) of patients with BC based on *ATP7B* expression levels. Patients were divided into two groups based on their median expression levels [15]. The data were accessed on May 3, 2021.

### Statistical analyses

Numerical variables between the two groups were compared using the Mann–Whitney test; comparisons between multiple groups were performed using ANOVA followed by Tukey’s post hoc test. The correlation between *ATP7B* and cancer-related gene expression levels in PCR array analysis was assessed using Spearman’s rank correlation test. The associations between mRNA or protein expression levels of ATP7B and clinicopathological factors were analyzed using the χ^2^ test. Disease-free survival (DFS) and OS were calculated using the Kaplan–Meier method, and survival curves were compared using the log-rank test. Multivariate analysis was performed using the Cox hazard model. All statistical analyses were performed using JMP 16 software (SAS Institute Inc., Cary, NC, USA), and statistical significance was defined as *p* < 0.05.

## Results

### *ATP7B* mRNA expression and its association with other cancer-related genes in BC cell lines

**Fig. 1:**
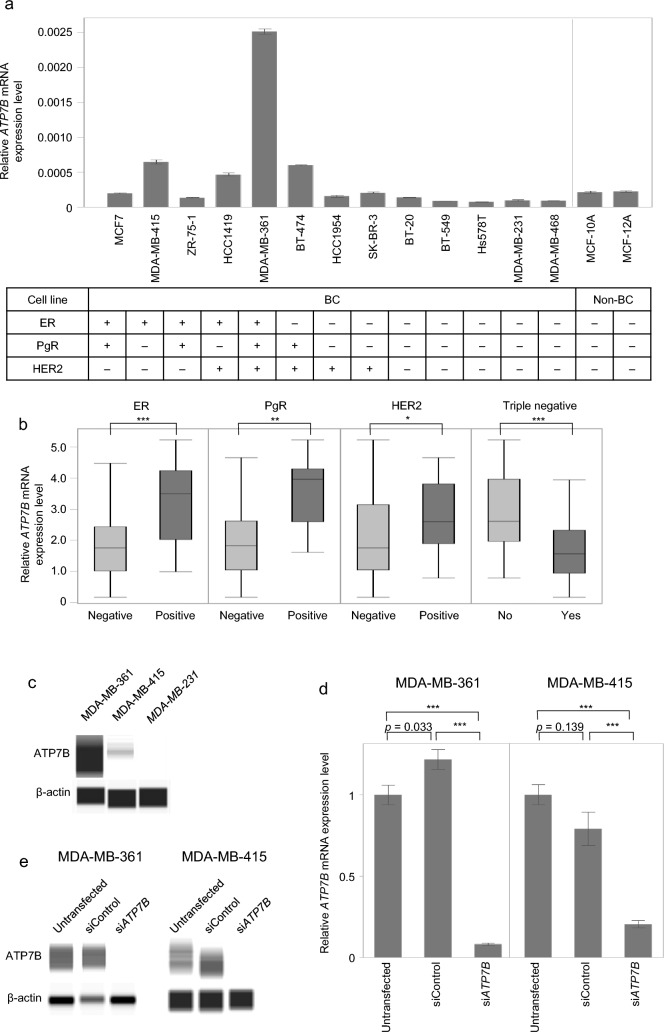
ATP7B expression in BC cell line. **a** *ATP7B* mRNA expression in 13 BC and two non-cancerous cell lines. Error bars, mean ± SEM. **b** Association between *ATP7B* mRNA expression levels and the status of conventional biomarkers from CCLE data. ER-positive cells, PgR-positive cells, and HER2-positive cells had significantly higher *ATP7B* mRNA levels than their negative counterparts. **c** ATP7B expression in representative BC cell lines. ATP7B expression was detected in MDA-MB-361 and MDA-MB-415, whereas ATP7B was not detected in MDA-MB-231 cells. **d** qRT-PCR analysis of *ATP7B* mRNA expression levels after knockdown in MDA-MB-361 and MDA-MB-415 cell lines. **e** Western blot analysis revealed inhibition of ATP7B after knockdown in MDA-MB-361 and MDA-MB-415 cell lines. *ATP7B* ATPase copper transforming beta, *BC* breast cancer, *ER* estrogen receptor, *HER2* human epidermal growth factor receptor 2, *si* small interfering. *p < 0.05, **p < 0.01, ***p < 0.001

The mRNA expression levels of *ATP7B* in 13 BC cell lines and two non-cancerous cell lines are shown in Fig. 1a. The ER, PgR, and HER2 statuses of cell lines have been evaluated in previous studies [16, 17]. *ATP7B* mRNA levels in ER-positive and HER2-positive cell lines were significantly higher than those in ER-negative and HER2-negative BC cells (*p* = 0.015 and *p* = 0.028, respectively). To compensate for the small number of cell lines, *ATP7B* mRNA expression levels in additional BC cell lines were obtained from the CCLE database for verification. The ER, PgR, and HER2 statuses of each cell line were obtained from previous studies [18–20]. Among the 60 BC cells with available data regarding *ATP7B* expression levels, ER-positive, PgR-positive, and HER2-positive cells had significantly higher *ATP7B* mRNA levels than their negative counterparts (*p* < 0.001, *p* = 0.002, and *p* = 0.040, respectively; Fig. 1b).

PCR array analysis revealed that *ATP7B* mRNA expression levels were positively correlated with those of several well-known oncogenes, such as *cadherin 1* (*CDH1*) and *estrogen receptor 1* (*ESR1*), and negatively correlated with *MET proto-oncogene* (*MET*) (Tables 1 and S1).

**Table 1:** Correlations between mRNA expression levels of *ATP7B* and cancer-related genes

Gene	Official full name	Correlation coefficient	*p*-value
*CDH1*	Cadherin 1, type 1, E-cadherin (epithelial)	0.863	< 0.001
*ESR1*	Estrogen receptor 1	0.709	0.007
*RET*	Ret proto-oncogene	0.681	0.010
*ZHX2*	Zinc fingers and homeoboxes 2	0.632	0.021
*MYB*	V-myb myeloblastosis viral oncogene homolog (avian)	0.615	0.025
*MYCN*	V-myc myelocytomatosis viral related oncogene, neuroblastoma derived	0.615	0.025
*MET*	Met proto-oncogene (hepatocyte growth factor receptor)	− 0.681	0.010
*JUN*	Jun proto-oncogene, AP-1 transcription factor subunit	− 0.615	0.025
*ETS1*	V-ets erythroblastosis virus E26 oncogene homolog 1 (avian)	− 0.610	0.027
*PML*	Promyelocytic leukemia	− 0.593	0.033
*TGFB1*	Transforming growth factor, beta 1	− 0.566	0.044

### Effects of ATP7B knockdown in BC cell lines

Western blotting was performed using representative BC cell lines with high or low *ATP7B* mRNA expression to confirm the protein expression of ATP7B. Among these cell lines, MDA-MB-361 represents the ER-positive/HER2-positive subtype, and MDA-MB-415 represents the ER-positive/HER2-negative subtype. MDA-MB-231, one of the cell lines with the lowest *ATP7B* mRNA expression, was used as a negative control (Fig. 1c). Cells transfected with siRNA expressed lower levels of ATP7B mRNA and protein (Fig. 1d and e).

**Fig. 2:**
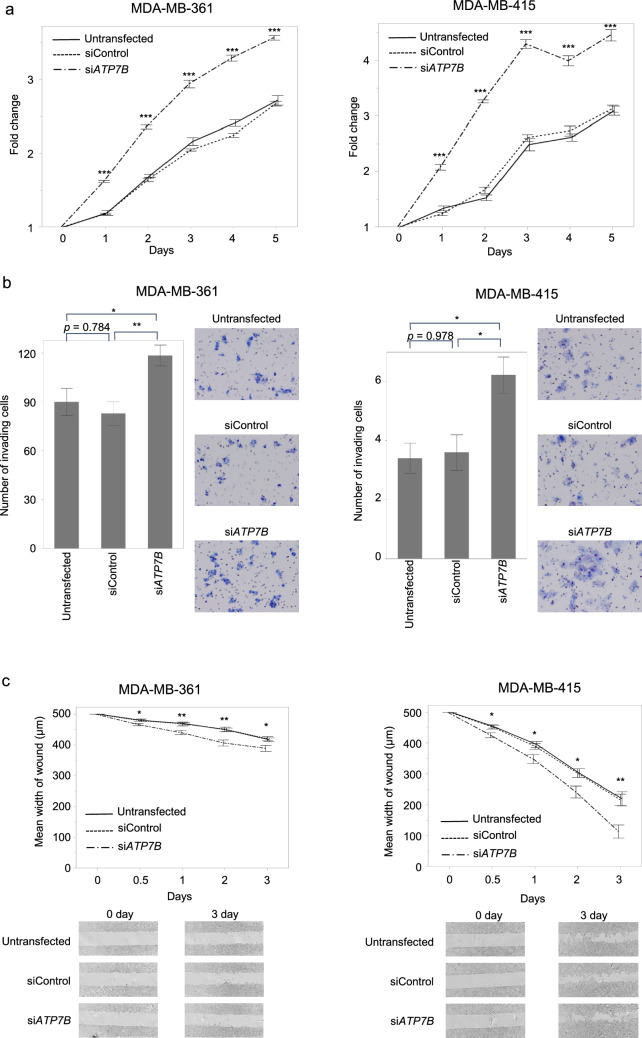
Functional analysis in BC cell lines using knockdown cells. **a** Proliferation assay: si*ATP7B*-transfected cell proliferation was significantly enhanced compared to untransfected and siControl-transfected cells. **b** Invasiveness assay: ATP7B knockdown in BC cells significantly increased the number of invading cells. **c** Migration assay: the migration ability of MDA-MB-361 and MDA-MB-415 cells was enhanced after si*ATP7B* transfection. Error bars mean ± SEM. *ATP7B* ATPase copper transporting beta, *BC* breast cancer, *si* small interfering. *p < 0.05, **p < 0.01, ***p < 0.001

To determine the oncological role of ATP7B in BC cells, cell proliferation, invasiveness, and migration were evaluated using knockdown cells. During the entire study period, proliferation was significantly enhanced in si*ATP7B*-transfected MDA-MB-361 and MDA-MB-415 cells compared to that in untransfected and siControl-transfected cells (Fig. 2a). In the invasiveness assay, more si*ATP7B*-transfected than siControl-transfected or untransfected MDA-MB-361 and MDA-MB-415 cells passed through the Matrigel (Fig. 2b). Moreover, the migratory abilities of MDA-MB-361 and MDA-MB-415 cells were enhanced following si*ATP7B* transfection (Fig. 2c).

### Association between *ATP7B* mRNA expression levels and clinicopathological factors

**Fig. 3:**
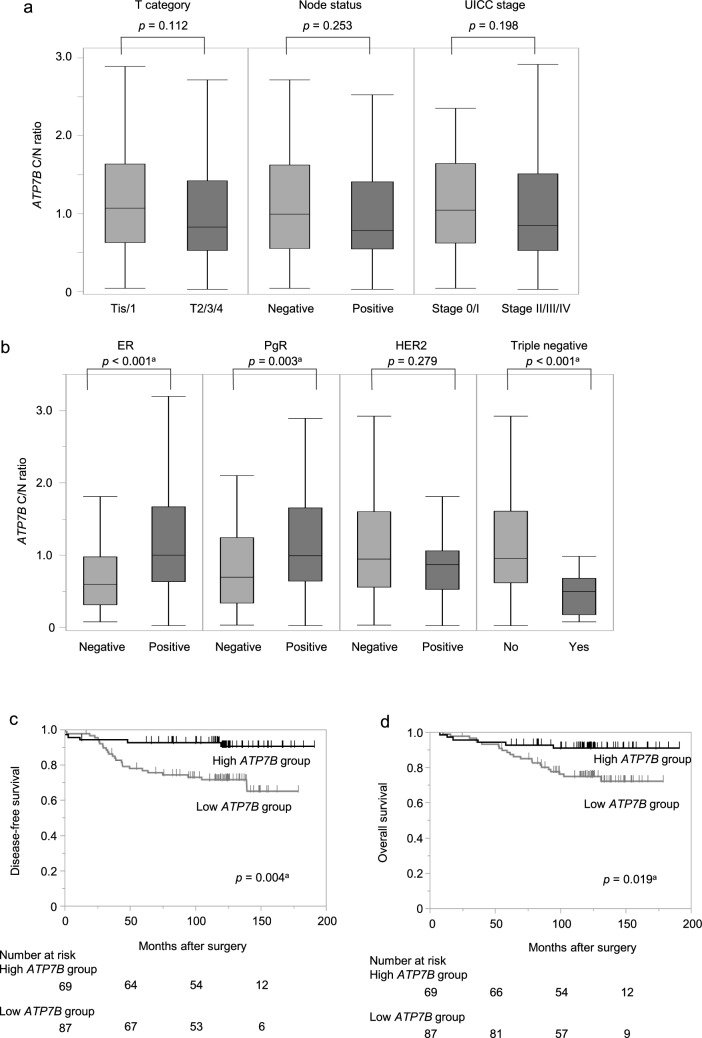
Association between ATP7B mRNA expression levels and clinicopathological factors. **a** *ATP7B* mRNA expression levels did not differ according to T-category, lymph node metastasis, or UICC stage. **b** ER-positive and PgR-positive samples had significantly higher *ATP7B* mRNA expression levels than ER-negative and PgR-negative samples; however, no significant difference was found between HER2-positive and HER2-negative specimens. **c** High *ATP7B* group experienced longer DFS than the low *ATP7B* group. **d** The OS rates in the high *ATP7B* group were significantly longer than those in the low *ATP7B* group. ^a^*p* < 0.05; *ATP7B* ATPase copper transporting beta, *DFS* disease‑free survival, *ER* estrogen receptor, *HER2* human epidermal growth factor receptor 2, *OS* overall survival, *PgR* progesterone receptor, *Tis* carcinoma *in situ*, *UICC* Union for International Cancer Control

*ATP7B* mRNA expression levels were evaluated in both BC and non-cancerous specimens. The ratio of *ATP7B* mRNA expression levels between cancerous and non‑cancerous specimens was defined as the ‘C/N ratio.’ The mean C/N ratio (± SD) was 1.28 ± 1.64, with 69 (44.2%) patients having a C/N ratio greater than one. The *ATP7B* C/N ratios were not predominant in the T category, lymph node metastasis, or UICC stage (Fig. 3a). Regarding conventional biomarkers, ER-positive specimens (n = 119) had a higher *ATP7B* C/N ratio than ER-negative specimens (n = 37; *p* < 0.001), and PgR-positive specimens (n = 108) had significantly higher *ATP7B* C/N ratios than PgR-negative specimens (n = 48; *p* = 0.003; Fig. 3b). The *ATP7B* C/N ratio did not differ significantly between HER2-positive (n = 37) and HER2-negative specimens (n = 111; *p* = 0.279; Fig. 3b).

Patients with a C/N ratio greater than one were assigned to the “high *ATP7B* group” (n = 69), while those with a C/N ratio less than one were assigned to the “low *ATP7B* group” (n = 87). The associations between clinicopathological factors and *ATP7B* expression are shown in Table 2. Tumor size, lymph node metastasis, or UICC pathological stage did not differ significantly between the two groups. The high *ATP7B* group had more ER-positive and PgR-positive patients than the low *ATP7B* group (*p* = 0.002 and *p* = 0.030, respectively).

**Table 2:** Associations between *ATP7B* mRNA expression and the clinicopathological characteristics of 156 patients with breast cancer

Characteristics	Expression of *ATP7B*		*p*-value
	High *ATP7B* group (n = 69)	Low *ATP7B* group (n = 87)	
Age (range)	50 (26–78)	54 (30–77)	0.024^a^
Histology			0.138
DCIS	5 (7.3%)	1 (1.1%)	
IDC	57 (82.6%)	81 (93.1%)	
ILC	4 (5.8%)	2 (2.3%)	
Other	3 (4.3%)	3 (3.5%)	
UICC T category			0.050
Tis/T1	37 (53.6%)	33 (37.9%)	
T2/T3/T4	32 (46.4%)	54 (62.1%)	
Lymph node status			0.147
Positive	30 (43.5%)	48 (55.2%)	
Negative	39 (56.5%)	39 (44.8%)	
UICC pathological stage			0.133
0/I	26 (37.7%)	23 (26.4%)	
II/III/IV	43 (62.3%)	64 (73.6%)	
ER status			0.002^a^
Positive	61 (88.4%)	58 (66.7%)	
Negative	8 (11.6%)	29 (33.3%)	
PgR status			0.030^a^
Positive	54 (78.3%)	54 (62.1%)	
Negative	15 (21.7%)	33 (37.9%)	
HER2 status			0.176
Positive	12 (17.4%)	25 (28.7%)	
Negative	52 (75.4%)	59 (67.8%)	
Unknown	5 (2.2%)	3 (3.5%)	
Adjuvant therapy			0.101
Endocrine therapy alone	28 (40.6%)	22 (25.3%)	
Chemotherapy alone	8 (11.6%)	21 (24.1%)	
Endocrine and chemotherapy	26 (37.7%)	36 (41.4%)	
None	7 (10.1%)	8 (9.2%)	

Data are expressed as the median (range) or number (%)

*ATP7B* ATPase copper transporting beta, *DCIS* ductal carcinoma *in situ*, *ER* estrogen receptor, *HER2* human epidermal growth factor 2, *IDC* invasive ductal carcinoma, *ILC* invasive lobular carcinoma, *PgR* progesterone receptor, *Tis* tumor *in situ*, *UICC* Union for International Cancer control

^a^*p* < 0.05

The high *ATP7B* group had a significantly longer DFS than the low *ATP7B* group (5-year DFS, high *ATP7B* group: 92.7%, low *ATP7B* group: 76.6%; *p* = 0.004; Fig. 3c). The OS rates in the high *ATP7B* group were also longer than that in the low *ATP7B* group (5-year OS: high *ATP7B* group, 92.8%; low *ATP7B* group, 88.4%; *p* = 0.019; Fig. 3d). To compensate for the small number of patients in our cohort, the prognostic value of *ATP7B* expression was validated using the Kaplan–Meier plotter website. Similarly, when patients were separated based on the median *ATP7B* expression, the high *ATP7B* expression group exhibited significantly longer RFS (n = 4929; *p* < 0.001) and OS (n = 1879; *p* = 0.013) (Fig.S1 a and b). Multivariate analysis of DFS revealed ‘lymph node metastasis’ (HR, 3.56; 95% CI 1.42–8.92; *p* = 0.007) and ‘low *ATP7B* expression’ (HR, 2.82; 95% CI 1.15–6.92; *p* = 0.024) as independent prognostic factors (Table 3).

**Table 3:** Prognostic factors for disease-free survival in 156 breast cancer patients

Variable	n	Univariate			Multivariate		
		Hazard ratio	95% CI	*p*-value	Hazard ratio	95% CI	*p*-value
Age, > 60 years	53	1.03	0.48–2.18	0.948			
Tumor size, > 2 cm	86	3.69	1.51–8.99	0.004^a^	2.27	0.91–5.68	0.081
Node status, positive	78	4.99	2.05–12.2	< 0.001^a^	3.56	1.42–8.92	0.007^a^
ER status, negative	37	1.82	0.86–3.88	0.120			
PgR status, negative	48	1.59	0.77–3.27	0.210			
HER2 status, positive	37	1.93	0.92–4.06	0.082			
Low *ATP7B* expression	87	3.66	1.50–8.93	0.004^a^	2.82	1.15–6.92	0.024^a^

Univariate analysis: Cox proportional hazards model. Multivariate analysis: Cox proportional hazards model

*CI* confidence interval, *ER* estrogen receptor, *HER2* human epidermal growth factor 2, *PgR* progesterone receptor

^a^*p* < 0.05

### Assessment of ATP7B protein expression status by immunohistochemistry

**Fig. 4:**
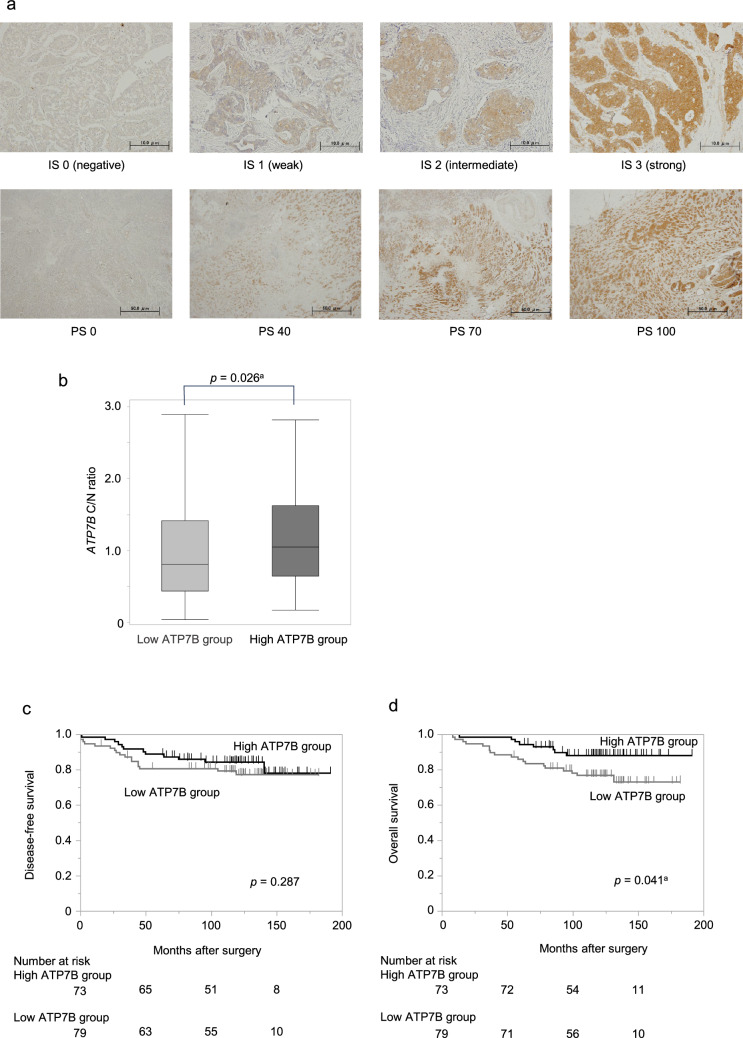
Assessment of ATP7B protein expression status by immunohistochemistry. **a** Representative staining for the IS and PS of ATP7B. **b** *ATP7B* C/N ratio was higher in the high ATP7B group than in the low ATP7B group. **c** No difference in DFS was found. **d** The OS rates in the high ATP7B group were significantly longer than those in the low ATP7B group. ^a^*p* < 0.05; *ATP7B* ATPase copper transporting beta, *DFS* disease‑free survival, *IS* intensity of staining, *OS* overall survival, *PS* percentage of staining

At the protein level, cytoplasmic ATP7B staining was evaluated by immunohistochemistry in 152 BC specimens. Representative staining of the IS and PS of ATP7B is shown in Fig. 4a. Patients with an IP score (IS x PS score) of 150 or higher were assigned to the ‘high ATP7B group’ (n = 73) while the remaining patients were assigned to the ‘low ATP7B group’ (n = 79). The *ATP7B* C/N ratio was higher in the high ATP7B group than in the low ATP7B group (*p* = 0.026; Fig. 4b), which validated the consistency between the mRNA and protein levels. Although DFS did not differ (Fig. 4c), the OS rates were significantly higher in the high ATP7B group than in the low ATP7B group (5-year OS: high ATP7B group, 94.5%; low ATP7B group, 86.1%; *p* = 0.041; Fig. 4d). Multivariate analysis of OS revealed ‘lymph node metastasis’ (HR, 4.71; 95% CI 1.75–12.7; *p* = 0.002), ‘ER negative’ (HR, 7.92; 95% CI 1.01–61.8; *p* = 0.048), and ‘low ATP7B expression’ (HR, 2.38; 95% CI 1.02–5.57; *p* = 0.046) as independent prognostic factors (Table S2).

## Discussion

In this study, ATP7B interfered with tumor progression by suppressing the proliferation, invasiveness, and migration of BC cells. Furthermore, the analysis of clinical specimens revealed that ATP7B mRNA and protein expression were independent prognostic factors, thus supporting the *in vitro* results.

ATP7B is a member of the P-type ATPase family and is involved in intracellular copper transport and homeostasis [3]. Notably, *ATP7B* is one of the genes that has attracted considerable attention for its potential involvement in cuproptosis [6]. According to a recent study, *ATP7B* is differentially expressed in various carcinomas, suggesting a prognostic implication for patients with low-grade glioma and renal clear cell carcinoma [21]. *ATP7B* may also serve as a therapeutic target to improve the efficacy of docetaxel in prostate cancer [22]. In BC, *ATP7B* induces resistance to cisplatin [9]. High *ATP7B* expression in patients with ER-positive BC has also been reported to be associated with a lower risk of relapse [23]. However, the oncological role and significance of ATP7B in patients have not been elucidated to date.

In this study, *ATP7B* was highly expressed in ER-positive cell lines, consistent with the results obtained from the CCLE database. Furthermore, our clinical specimens from ER-positive patients with BC were found to have high expression of *ATP7B* compared to those from ER-negative patients. Based on PCR array analysis, the expression level of *ESR1* correlated positively with that of *ATP7B*, thus supporting these observational results. A previous study did not find a significant relationship between *ATP7B* and ER-positivity in patients with BC [9]; this finding may be due to the small number of samples (41 patients), which resulted in insufficient statistical power. The consistency of our results with cell lines and clinical samples implies that ATP7B is involved in the ER signaling pathway in BC. However, further pathway analyses are required to clarify the role of ATP7B in this regard.

Overall, knockdown of ATP7B was found to promote malignant phenotypes in BC cells and patients with high ATP7B expression had a favorable prognosis in this study. The expression status of ATP7B was also identified as an independent prognostic factor at both the mRNA and protein levels, leading to more robust results. Although functional analysis of ATP7B in BC cells and the prognostic impact of its expression in patients with BC have not been previously assessed, a recent study revealed the association between cuproptosis-related gene expression and prognosis in ER-positive patients with BC [23]. Accordingly, patients with high expression of some cuproptosis-related genes, including *ATP7B*, had favorable relapse-free survival rates. Although these results are similar to those of our study, we have demonstrated that ATP7B alone can be used as a prognostic marker for BC, regardless of subtypes.

Although functional analyses revealed that ATP7B plays tumor-suppressive roles in BC cells, elucidating the underlying mechanism remains a difficult task. Nevertheless, based on the results of our PCR array analysis of BC cells and those of previous studies, several explanations can be proposed. The highest correlation coefficient was observed between the expression levels of *ATP7B* and *CDH1*, which encodes E-cadherin. E-cadherins contribute to the establishment and maintenance of polarized and differentiated epithelia through intercellular adhesion complexes, and their inactivation increases the metastatic capacity, which leads to poor prognoses in BC [24]. In contrast, *ATP7B* expression negatively correlated with *MET* expression. *MET* encodes the tyrosine kinase receptor for hepatocyte growth factor, and its activation promotes cellular invasiveness, angiogenesis, and metastasis [25]. Although no studies to date have suggested a direct relationship between ATP7B and these molecules, ATP7B may affect the expression of these genes to suppress tumor progression in BC.

Notably, analysis of the clinical samples identified both the mRNA and protein levels of ATP7B as prognostic markers. The cut-off values of ATP7B expression levels have not been established to date. In this study, in terms of the mRNA expression levels, patients with a C/N ratio greater than one were assigned to the “high *ATP7B* group” (n = 69) while those with a C/N ratio less than one were assigned to the “low *ATP7B* group” (n = 87). This grouping method is considered relatively reasonable. On the other hand, for the immunohistochemistry, we grouped the patients according to the IP score. Patients with an IP score of 150 or higher were then assigned to the ‘high ATP7B group’ (n = 73) while the remaining patients were assigned to the ‘low ATP7B group’ (n = 79). This cut-off value allocated almost the same number of cases as the grouping at the mRNA expression levels. Moreover, because *ATP7B* mRNA expression was shown to be higher in ‘high ATP7B group’, this criterion closely reflected the grouping at the mRNA level. Although our results are supported by the public database, further validation studies will be required to determine appropriate cut-off values.

This study had several limitations. As described above, the mechanism by which ATP7B is involved in tumor suppression has not been fully investigated, despite the proposal of several theories. In particular, the mechanism by which ATP7B affects copper transport in BC cells to attenuate their malignant features remains to be elucidated. Second, adjuvant medication therapy could have affected the patient prognoses in our cohort data. To compensate for this effect, we used a public database to validate our results. In addition, further studies, such as *in vivo* experiments, are warranted to elucidate the potential therapeutic targets of ATP7B.

In conclusion, this study revealed the tumor-suppressive roles of ATP7B in BC cells. Moreover, this study highlighted that high ATP7B expression in cancerous tissues could be a favorable prognostic biomarker in patients with BC.

## Supplementary Information

Below is the link to the electronic supplementary material.

Supplementary Fig1 Prognosis according to ATP7B expression levels from the Kaplan-Meier plotter website. a. Higher ATP7B expression group had a significantly longer RFS than the lower ATP7B expression group. b. Higher ATP7B expression group had a significantly longer OS than the lower ATP7B expression group. a p0.05; ATP7B, ATPase copper transporting beta; RFS, relapse-free survival; OS, overall survival
Supplementary file1 (PDF 81 KB)

Supplementary file2 (PDF 223 KB)

Supplementary file3 (PDF 120 KB)

## Data Availability

The datasets used and/or analyzed in the current study are available from the corresponding author upon reasonable request.
