# Supplementary material for: ATPase copper transporting beta attenuates malignant features with high expression as an indicator of favorable prognosis in breast cancer
Source: Breast Cancer. 2025 May 2;32(4):803–15. doi: 10.1007/s12282-025-01705-7 (PMC12174277; doi:10.1007/s12282-025-01705-7)
Supplement: Supplementary file 1 — Supplementary Fig1 Prognosis according to ATP7B expression levels from the Kaplan-Meier plotter website. a. Higher ATP7B expression group had a significantly longer RFS than the lower ATP7B expression group. b. Higher ATP7B expression group had a significantly longer OS than the lower ATP7B expression group. a p0.05; ATP7B, ATPase copper transporting beta; RFS, relapse-free survival; OS, overall survival Supplementary file1 (PDF 81 KB) [file 12282_2025_1705_MOESM1_ESM.pdf]

Supplementary Figure1

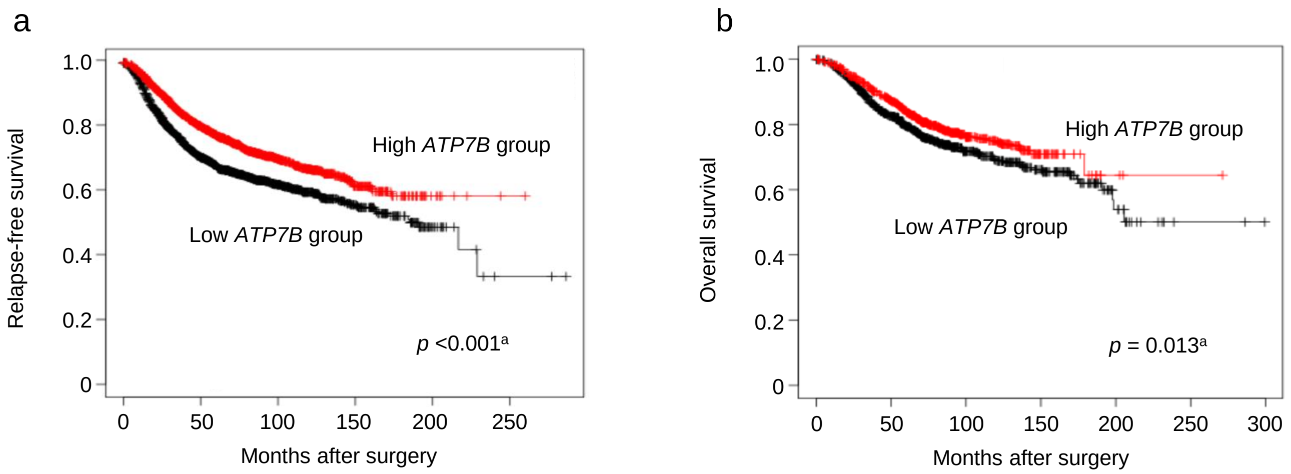

a

Number at risk

High *ATP7B* group	2464	1558	622	116	9	1
Low *ATP7B* group	2465	1325	514	130	18	2

b

Number at risk

High *ATP7B* group	937	729	292	43	3	1	0
Low *ATP7B* group	942	637	261	100	18	2	0
