# Supplementary material for: ATPase copper transporting beta attenuates malignant features with high expression as an indicator of favorable prognosis in breast cancer
Source: Breast Cancer. 2025 May 2;32(4):803–15. doi: 10.1007/s12282-025-01705-7 (PMC12174277; doi:10.1007/s12282-025-01705-7)
Supplement: Supplementary file 2 — Supplementary file2 (PDF 223 KB) [file 12282_2025_1705_MOESM2_ESM.pdf]

Supplementary Table 1. Correlations between mRNA expression levels of *ATP7B* and 84 cancer-related genes.

Gene	Official Full Name	Correlation Coefficient	*p*-value
ABL1	ABL proto-oncogene 1, non-receptor tyrosine kinase	−0.110	0.721
AKT1	AKT serine/threonine kinase 1	0.132	0.668
APC	APC regulator of WNT signaling pathway	−0.077	0.803
ATM	ATM serine/threonine kinase	−0.214	0.482
BAX	BCL2 associated X, apoptosis regulator	−0.451	0.122
BCL2	BCL2 apoptosis regulator	0.176	0.566
BCL2L1	BCL2 like 1	−0.187	0.541
BCR	BCR activator of RhoGEF and GTPase	−0.115	0.707
BRCA1	BRCA1 DNA repair associated	−0.033	0.915
BRCA2	BRCA2 DNA repair associated	−0.066	0.831
CASP8	caspase 8	−0.280	0.354
CCND1	cyclin D1	0.456	0.117
CDH1	cadherin 1	0.863	<0.001
CDK4	cyclin dependent kinase 4	0.088	0.775
CDKN1A	cyclin dependent kinase inhibitor 1A	0.137	0.655
CDKN2A	cyclin dependent kinase inhibitor 2A	0.055	0.859
CDKN2B	cyclin dependent kinase inhibitor 2B	−0.325	0.279
CDKN3	cyclin dependent kinase inhibitor 3	−0.176	0.566
CTNNB1	catenin beta 1	−0.489	0.090
E2F1	E2F transcription factor 1	−0.412	0.162
EGF	epidermal growth factor	0.330	0.271
ELK1	ETS transcription factor ELK1	−0.148	0.629
ERBB2	erb-b2 receptor tyrosine kinase 2	0.495	0.086
ESR1	estrogen receptor 1	0.709	0.007
ETS1	ETS proto-oncogene 1, transcription factor	−0.610	0.027
FHIT	fragile histidine triad diadenosine triphosphatase	0.066	0.831
FOS	Fos proto-oncogene, AP-1 transcription factor subunit	0.093	0.762
FOXD3	forkhead box D3	−0.044	0.887
HGF	hepatocyte growth factor	0.055	0.858
HIC1	HIC ZBTB transcriptional repressor 1	−0.484	0.094
HRAS	HRas proto-oncogene, GTPase	−0.434	0.138
IGF2R	insulin like growth factor 2 receptor	−0.352	0.239
JAK2	Janus kinase 2	−0.385	0.194
JUN	Jun proto-oncogene, AP-1 transcription factor subunit	−0.132	0.668
JUNB	JunB proto-oncogene, AP-1 transcription factor subunit	−0.615	0.025

JUND	JunD proto-oncogene, AP-1 transcription factor subunit	−0.368	0.216
KIT	KIT proto-oncogene, receptor tyrosine kinase	0.478	0.099
KITLG	KIT ligand	0.302	0.316
KRAS	KRAS proto-oncogene, GTPase	0.379	0.201
MCL1	MCL1 apoptosis regulator, BCL2 family member	−0.006	0.986
MDM2	MDM2 proto-oncogene	0.396	0.181
MEN1	menin 1	−0.066	0.831
MET	MET proto-oncogene, receptor tyrosine kinase	−0.681	0.010
MGMT	O-6-methylguanine-DNA methyltransferase	0.170	0.578
MLH1	mutL homolog 1	−0.253	0.405
MOS	MOS proto-oncogene, serine/threonine kinase	0.126	0.681
MYB	MYB proto-oncogene, transcription factor	0.615	0.025
MYC	MYC proto-oncogene, bHLH transcription factor	−0.346	0.247
MYCN	MYCN proto-oncogene, bHLH transcription factor	0.615	0.025
NF1	neurofibromin 1	0.143	0.642
NF2	neurofibromin 2	−0.253	0.405
NFKB1	nuclear factor kappa B subunit 1	−0.028	0.929
NFKBIA	NFKB inhibitor alpha	0.379	0.201
NRAS	NRAS proto-oncogene, GTPase	−0.346	0.247
PIK3C2A	phosphatidylinositol-4-phosphate 3-kinase catalytic subunit type 2 alpha	0.187	0.541
PIK3CA	phosphatidylinositol-4,5-bisphosphate 3-kinase catalytic subunit alpha	−0.022	0.943
PML	PML nuclear body scaffold	−0.593	0.033
PRKCA	protein kinase C alpha	−0.506	0.078
RAF1	Raf-1 proto-oncogene, serine/threonine kinase	-0.258	0.394
RARA	retinoic acid receptor alpha	0.335	0.263
RASSF1	Ras association domain family member 1	−0.506	0.078
RB1	RB transcriptional corepressor 1	0.302	0.316
REL	REL proto-oncogene, NF-kB subunit	−0.121	0.694
RET	ret proto-oncogene	0.681	0.010
ROS1	ROS proto-oncogene 1, receptor tyrosine kinase	−0.286	0.344
RUNX1	RUNX family transcription factor 1	−0.093	0.762
RUNX3	RUNX family transcription factor 3	−0.055	0.859
S100A4	S100 calcium binding protein A4	−0.236	0.437
SERPINB5	serpin family B member 5	0.451	0.122
SH3PXD2A	SH3 and PX domains 2A	−0.484	0.094
SMAD4	SMAD family member 4	0.060	0.845
SRC	SRC proto-oncogene, non-receptor tyrosine kinase	−0.176	0.566
STAT3	signal transducer and activator of transcription 3	−0.412	0.162

STK11	serine/threonine kinase 11	−0.434	0.139
TGFB1	transforming growth factor beta 1	−0.566	0.044
TNF	tumor necrosis factor	−0.478	0.099
TP53	tumor protein p53	−0.467	0.108
TP73	tumor protein p73	0.341	0.255
TSC1	TSC complex subunit 1	−0.071	0.817
VHL	von Hippel-Lindau tumor suppressor	0.044	0.887
WT1	WT1 transcription factor	−0.154	0.616
WWOX	WW domain containing oxidoreductase	0.297	0.325
XRCC1	X-ray repair cross complementing 1	−0.044	0.887
ZHX2	zinc fingers and homeoboxes 2	0.632	0.021
