# Supplementary material for: ATPase copper transporting beta attenuates malignant features with high expression as an indicator of favorable prognosis in breast cancer
Source: Breast Cancer. 2025 May 2;32(4):803–15. doi: 10.1007/s12282-025-01705-7 (PMC12174277; doi:10.1007/s12282-025-01705-7)
Supplement: Supplementary file 3 — Supplementary file3 (PDF 120 KB) [file 12282_2025_1705_MOESM3_ESM.pdf]

Supplementary Table 2. Prognostic factors for overall survival in 152 breast cancer patients.

		Univariate			Multivariate		
Variable	n	Hazard ratio	95% CI	*p*-value	Hazard ratio	95% CI	*p*-value
Age, >60 years	50	1.56	0.72–3.36	0.260			
Tumor size, >2cm	82	4.05	1.53–10.7	0.005[a]	2.63	0.95–7.29	0.063
Node status, positive	74	5.40	2.04–14.3	<0.001[a]	4.71	1.75–12.7	0.002[a]
ER status, negative	37	3.51	1.65–7.48	0.001[a]	7.92	1.01–61.8	0.048[a]
PgR status, negative	47	2.77	1.30–5.89	0.008[a]	0.35	0.04–2.81	0.324
HER2 status, positive	36	2.02	0.92–4.45	0.082			
Low ATP7B expression	79	2.34	1.03–5.35	0.044[a]	2.38	1.02–5.57	0.046[a]

[a]$p<0.05$. Univariate analysis: Cox proportional hazards model. Multivariate analysis: Cox proportional hazards model. CI, confidence interval; ER, estrogen receptor; HER2, human epidermal growth factor 2; PgR, progesterone receptor
